# The efficacy and safety of Gukang Capsule for primary osteoporosis: a systematic review and meta-analysis of randomized clinical trial

**DOI:** 10.3389/fphar.2024.1394537

**Published:** 2024-06-10

**Authors:** Zhenpu Wei, Zhiqiang Wang, Yunmei Huang, Xuzheng Chen, Pan Sun, Chutian Zhang, Fen Zhou, Yanping Lin

**Affiliations:** ^1^ Academy of Integrative Medicine, Fujian University of Traditional Chinese Medicine, Fuzhou, Fujian, China; ^2^ School of Acupuncture-Moxibustion and Tuina, Fujian University of Traditional Chinese Medicine, Fuzhou, Fujian, China; ^3^ Fujian Key Laboratory of Integrative Medicine on Geriatrics, Fuzhou, Fujian, China

**Keywords:** primary osteoporosis, Gukang Capsule, systematic review, complementary and alternative medicine, randomized clinical trial

## Abstract

**Background:** Gukang Capsule has been used as a complementary and alternative medicine (CAM) for the treatment of primary osteoporosis (POP) in China. The primary aim of this study was to assess the clinical effectiveness and safety of Gukang Capsule in POP patients.

**Methods:** A systematic search was conducted across multiple academic databases including PubMed, Web of science, Cochrane Library, China National Knowledge Infrastructure, Chongqing VIP Information, and Wanfang database to identify randomized controlled trials investigating the Gukang Capsule in the treatment of POP. The screening process, data extraction, and assessment of methodological quality were conducted independently by two reviewers. Statistical analysis was performed using the Rev Man 5.3 software. Subgroup analysis was carried out through the combination of OPF. Subgroup analysis was performed according to whether OPF were combined. Stata 12.0 was used for sensitivity and bias analysis.

**Results:** Nineteen studies were assessed that included 1804 participants. It was found that compared with the control group, the total effective rate (RR = 1.26, 95% CI, 1.20, 1.33), the Medical Outcomes Study Short-form 36 [RR = 1.26, 95% CI(1.20, 1.33)], the bone mineral density (BMD) of lumbar vertebra (SMD = 0.77, 95% CI, 0.48, 1.07), the BMD of femoral neck [SMD = 0.84, 95% CI(0.53, 1.14)], and the BMD of Ward’s triangle (SMD = 0.64, 95% CI, 0.44, 0.85) of the Gukang Capsule experimental group were higher. Compared with the control group, the fracture healing time (SMD = −2.14, 95% CI, −2.45, −1.84), the bone specific alkaline phosphatase (BALP) levels in serum (SMD = −2.00, 95% CI, −2.83, −1.17), the tartrate resistant acid phosphatase 5b (TRACP-5b) levels in serum (SMD = −2.58, 95% CI, −3.87, −1.29) of the Gukang Capsule experimental group were lower. The bone glaprotein (BGP) levels in serum (SMD = −0.22, 95% CI, −1.86, 1.43) and the adverse events (RR = 0.80, 95% CI, 0.40, 1.63) of the experimental group and the control group have no difference.

**Conclusion:** Gukang Capsule, as a CAM for the management of POP, exhibits the potential to enhance BMD and quality of life, expedite the healing time of OPF, diminish levels of BALP and TRACP-5b, and improve the total effective rate without increasing the adverse events.

**Systematic Review Registration:** https://www.crd.york.ac.uk/prospero/display_record.php?ID=CRD42023477774, PROSPERO CRD42023477774.

## 1 Introduction

Osteoporosis (OP) is a metabolic bone disease characterized by loss and decrease of bone mass, destruction of bone tissue microstructure, increased brittleness, and reduced flexibility predisposes to an increased risk of fractures (Johnston and Dagar, 2020). The main clinical manifestations of OP are joint pain in the lower back and extremities, often accompanied by pathological fractures (Anthamatten and PArish, 2019). The prevalence of OP has been on the rise, and it is associated with multiple complications and high recurrence and mortality rates. In recent years, the incidence of OP has gradually increased, especially among middle-aged and elderly individuals (Panahi et al., 2023). Primary osteoporosis (POP), a physiologic degeneration associated with aging [including postmenopausal psteoporosis (PMOP) and senile osteoporosis (SOP)], is the most common type of OP (Amarnath et al., 2023).

Primary osteoporosis (POP) treatment typically involves medications such as calcium, sex hormones, bisphosphonates, calcitonin, and fluoride (Qaseem et al., 2023). However, these approaches can be limited by adverse drug reactions, patient intolerances, and uncertainties about the long-term efficacy of some medications. In recent years, Traditional Chinese Medicine (TCM) has emerged as a promising alternative, with some proprietary Chinese medicines gaining popularity among doctors and patients due to their perceived ease of use, consistent therapeutic effects, and minimal side effects (Li et al., 2022; Jia et al., 2023).

Gukang Capsule is a TCM preparation combining a Miao folk prescription from Guizhou with modern pharmaceutical technology. It is currently listed in the National Medical Products Administration Standard of the People’s Republic of China [WS-10464 (ZD-0464) -2005–2012Z] as a Class B variety of the National Medical Insurance (Zhu et al., 2022). Clinically, Gukang Capsule is widely used in treating orthopedic diseases because it is easy to use, stable, and has few side effects. Although there are currently many clinical studies on Gukang Capsule-based POP treatment, there is still a dearth of relevant evidence-based medical data on Gukang Capsule efficacy and safety (Liu, 2020). In 2022, the Orthopedics and Traumatology Branch of the Chinese Society of TCM formulated an ‘Expert Consensus on the Clinical Application of Gukang Capsule in the Treatment of Osteoporosis’ to guide clinicians on rational drug use. However, the lack of clinical evidence limits the usefulness of the available recommendations (Zhu et al., 2022). Against this background, we conducted a meta-analysis on the efficacy and safety of orally administered Gukang Capsule in POP treatment using evidence-based medical data and insights.

## 2 Materials and methods

The systematic review was registered in PROSPERO (CRD42023477774). To ensure accurate reporting of Gukang Capsule in this analysis, we followed the guidance established in the consensus statement on the Phytochemical Characterization of Medicinal Plant extract (ConPhyMP) (Supplementary Material S1–S4).

### 2.1 Search strategy

Randomized Controlled Trials (RCTs) of Gukang Capsule in POP treatment were searched in PubMed, Web of science, Cochrane Library, China National Knowledge Infrastructure, Chongqing VIP Information, and Wanfang databases. The retrieval time was from the databases’ inception to 30 September 2023. We adopted the retrieval strategy of combined subject words and free words. The keywords included: ‘Gukang Capsule’ ‘Gukang Jiaonang’ ‘Osteoporosis’ ‘Osteoporosis, Postmenopausal’ ‘Senile Osteoporosis’ and ‘Osteoporotic Fracture (OPF)’. The search process is detailed in the Supplementary Material S5.

### 2.2 Inclusion and exclusion criteria

#### 2.2.1 Inclusion criteria

(1) Study type: RCTs. (2) Participants: Patients definitively diagnosed with POP (PMOP, SOP, or OPF), bone mineral density (BMD) T value was ≤ −2.5 by dual-energy X-ray. (3) Control group: Any type of control group. (4) Experimental group: Treated with Gukang Capsule (orally administered) combined with other therapies. (5) Outcomes: Total effective rate, Short Form-36 Health Survey (SF-36), fracture healing time, BMD (lumbar vertebra, femoral neck, and Ward’s triangle), biochemical indices of serum bone metabolism [Bone Glaprotein (BGP), Bone Specific Alkaline Phosphatase (BALP), Tartrate Resistant Acid Phosphatase 5b (TRACP-5b)], and Adverse Events (AEs).

#### 2.2.2 Exclusion criteria

(1) Duplicate publications. (2) Unavailable full-text literature. (3) Studies with incomplete data.

### 2.3 Literature screening and data extraction

Literature search and screening were independently performed by two reviewers (Zhenpu Wei and Zhiqiang Wang). Specifically, they extracted basic information, intervention measures, outcomes, and other relevant data. A third reviewer (Yanping Lin) was consulted for any inconsistencies.

### 2.4 Quality assessment of the included studies

Two reviewers (Zhenpu Wei and Yunmei Huang) performed the biased risk assessment using the bias risk assessment tool recommended in the Cochrane Manual (Zhu et al., 2018). The details assessed were as follows: random sequence generation, allocation concealment, blinding of participants and personnel, blinding of outcome assessment, incomplete outcome data, selective reporting, and other relevant aspects. Each item was tagged as high risk, low risk, or unclear.

### 2.5 Statistical analysis

Clinical data were analyzed using Review Manager 5.3. Count data were evaluated using Relative Risk (RR) and 95% Confidence Interval (CI). Measurement data were analysed using the Standardised Mean Difference (SMD) and 95%CI (Huang et al., 2022). We further explored heterogeneity between the included studies. Studies were considered to have low variability if two conditions were met: 1) the I^2^ < 50%, and 2) the *p*-value >0.05. In cases with low variability, a fixed effects model was used to analyze the data. On the other hand, studies were considered to have high variability if either the I^2^ statistic was 50% or greater, or the *p*-value was greater than 0.05 (indicating a lack of statistical significance). When high variability was found, a random effects model was employed for data analysis (Cordero and Dans, 2020). Subgroup analyses were performed based on whether POP and OPF were combined. Sensitivity and bias analyses were performed using Stata 12.0 (Xu et al., 2009).

## 3 Results

### 3.1 Search results

**FIGURE 1:**
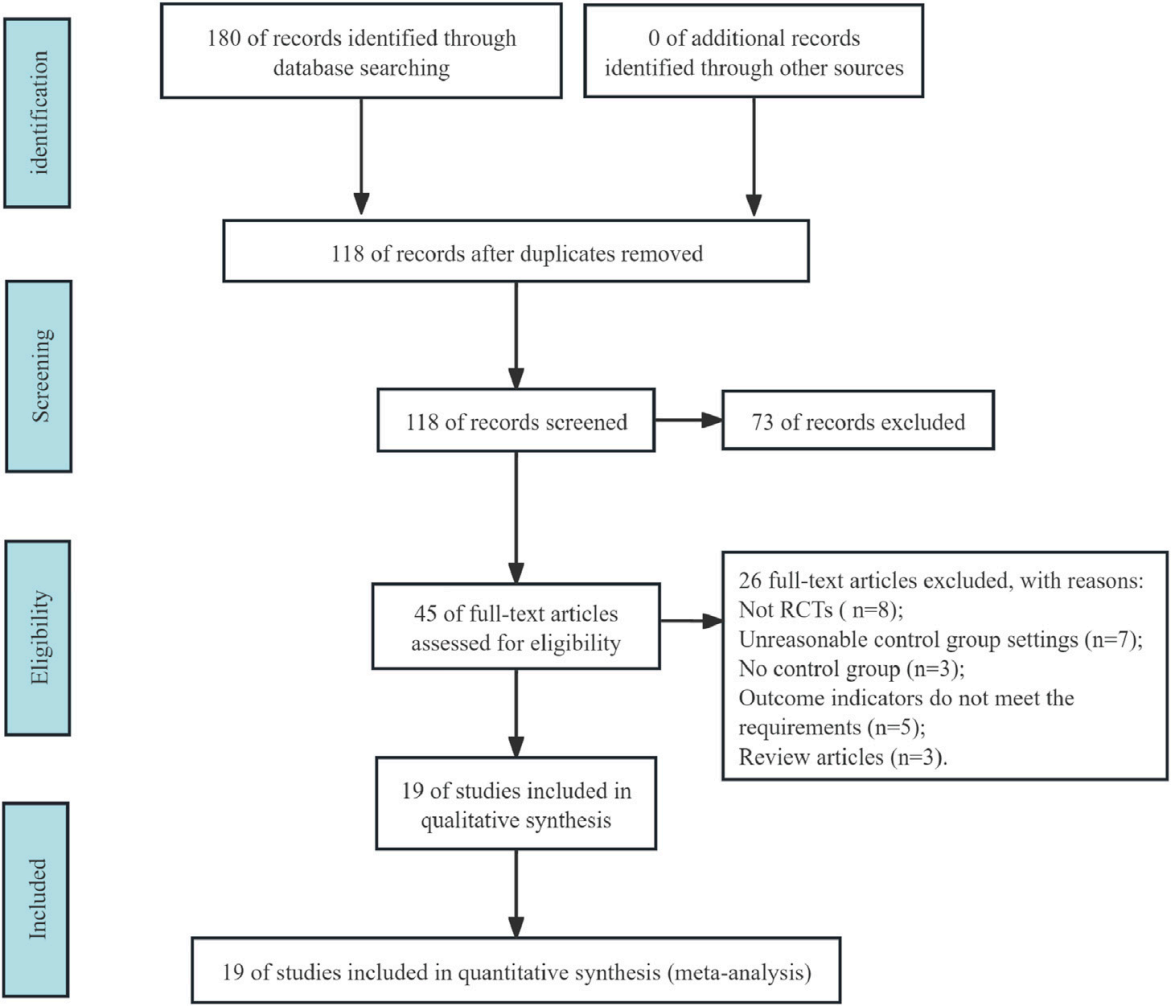
Flowchart of study selection.

Our initial search identified 180 articles. After removing duplicates (62 articles), we screened the abstracts of the remaining 118 articles. This process excluded 73 articles, leaving 45 full-text articles for further evaluation. Following a thorough review, 19 articles were ultimately determined to meet the inclusion criteria for this study. (Wang et al., 2014; Wang et al., 2015; Yu et al., 2015; Shen, 2016; Yan, 2016; Chen, 2018; Li, 2018; Li et al., 2019; Ye, 2019; Zhao et al., 2019; Liao et al., 2021; Yue and Huang, 2021; Zhou et al., 2021; Cai et al., 2022; Li et al., 2022; Xu, 2022; He et al., 2023; Sun and Xie, 2023; Tian, 2023) (Figure 1).

### 3.2 Characteristics of the included studies

Herein, 1804 POP patients (906 and 898 patients in the experimental and control groups, respectively) were included. Two studies (Chen, 2018; Ye, 2019) included POP patients, five studies (Li, 2018; Zhao et al., 2019; Li et al., 2022; Xu, 2022; Tian, 2023) included SOP patients, three studies (Wang et al., 2014; Yu et al., 2015; Liao et al., 2021) included PMOP patients, and nine studies (Wang et al., 2015; Shen, 2016; Yan, 2016; Li et al., 2019; Yue and Huang, 2021; Zhou et al., 2021; Cai et al., 2022; He et al., 2023; Sun and Xie, 2023) included POP+OPF patients. Except for one study (Cai et al., 2022), which did not mention the drug source, the other 18 studies (Wang et al., 2014; Wang et al., 2015; Yu et al., 2015; Shen, 2016; Yan, 2016; Chen, 2018; Li, 2018; Li et al., 2019; Ye, 2019; Zhao et al., 2019; Liao et al., 2021; Yue and Huang, 2021; Zhou et al., 2021; Li et al., 2022; Xu, 2022; He et al., 2023; Sun and Xie, 2023; Tian, 2023) mentioned that the Gukang Capsule used were produced by Guizhou Weikang Zifan Pharmaceutical Co., Ltd. (SINomatol: Z20025657) (Table 1).

**TABLE 1:** Characteristics of the included studies.

Study	Diagnosis	Sample size		Gender (male/female)		Age (years)		Intervention		Treatment duration (months)	Outcome
		CG	EG	CG	EG	CG	EG	CG	EG		
Chencheng 2018	POP	48	48	27/21	28/20	54.3 ± 3.4	55.2 ± 3.6	Salmon calcitonin injection	CG+Gukang Capsule	3	①④⑤⑥
Liguoqiang 2018	SOP	58	59	21/37	22/37	71.27 ± 7.70	71.44 ± 7.59	Alfacalcitol tablets	CG+Gukang Capsule	6	①②④⑦⑧⑨
Liaoxiaoyong 2021	PMOP	16	16	0/16	0/16	78.4 ± 3.2	78.1 ± 4.0	Alfacalcitol tablets	CG+Gukang Capsule	3	①
Liyonghua 2022	SOP	44	44	15/29	14/30	71.35 ± 4.67	71.44 ± 5.06	Alfacalcitol tablets	CG+Gukang Capsule	6	②④⑤⑦⑧⑩
Tianjiangping 2023	SOP	51	51	22/29	21/30	60.87 ± 6.89	61.11 ± 7.06	Calcium carbonate vitamin D3 tablets+Vitamin D3+Alendronate sodium tablets	CG+Gukang Capsule	12	①②⑦⑨
Wangbin 2014	PMOP	63	63	0/63	0/63	—	—	Calcium and vitamins	CG+Gukang Capsule	3	①④
Xuchong 2022	SOP	51	51	20/31	19/32	67.81 ± 5.80	67.88 ± 5.87	Caltrate D	CG+Gukang Capsule	6	①②④⑤⑥⑦⑧⑨⑩
Yuxianbin 2015	PMOP	30	30	0/30	0/30	59.63 ± 4.62	59.22 ± 5.05	Gukang capsule simulator+Caltrate D	CG+Gukang Capsule	6	①④⑤
Yechun 2019	POP	53	56	23/30	24/32	64 ± 5	64 ± 5	Calcium carbonate D3+Warm acupuncture	CG+Gukang Capsule	3	①④⑤⑧
Zhaojun 2019	SOP	40	40	21/19	18/22	71.6 ± 4.9	71.5 ± 5.2	Compound ossotide injection	CG+Gukang Capsule	6	①④⑤
Caimeihuang 2022	OPF (thoracolumbar spine)	58	60	31/27	35/25	64.31 ± 4.26	64.11 ± 4.07	Percutaneous kyphoplasty+Alendronate sodium tablets	CG+Gukang Capsule	6	①④⑤⑥⑧⑨
Hejianxing 2023	OPF (thoracolumbar spine)	39	39	20/19	22/17	69.05 ± 10.53	68.12 ± 10.48	Percutaneous kyphoplasty+Alendronate sodium tablets	CG+Gukang Capsule	1	①⑩
Lilin 2019	OPF (thoracolumbar spine)	56	56	25/31	22/34	65.5	67.1	Calcium carbonate D3	CG+Gukang Capsule	3	①
Shenruiwu 2016	OPF (distal radius)	41	41	19/22	17/24	52.8 ± 8.3	53.1 ± 8.6	Manual reduction or operation	CG+Gukang Capsule	—	③
Sunlei 2023	OPF (thoracolumbar spine)	50	50	27/23	29/21	63.38 ± 4.22	64.82 ± 4.65	Percutaneous vertebroplasty	CG+Gukang Capsule	12	①⑦
Wangwei 2015	OPF (distal radius)	54	54	24/30	25/29	65.38 ± 5.42	66.42 ± 5.35	T-plate internal fixation+Calcium carbonate tablets	CG+Gukang Capsule	2	③⑦
Yanyong 2016	OPF (distal radius)	36	36	12/24	11/25	66.6 ± 4.2	66.1 ± 5.5	Operated reduction+Calcium carbonate D3	CG+Gukang Capsule	6	③④⑤⑥⑦⑩
Yuehongwu 2021	OPF (hip)	49	51	15/34	19/32	66.37 ± 12.27	65.98 ± 12.29	Internal fixation +Calcitriol softgel capsules+Alendronate sodium tabiets+Caltrate D	CG+Gukang Capsule	6	④⑤⑦⑩
Zhouwei 2021	OPF (thoracolumbar spine)	61	61	33/28	30/31	46.86 ± 3.64	47.46 ± 4.23	Percutaneous vertebroplasty	CG+Gukang Capsule	2	①

Abbreviations: EG, experimental group; CG, Control Group; /: = not mentioned; POP, primary osteoporosis; PMOP, postmenopause osteoporosis; OPF, osteoporotic fracture; and SOP, Senile Osteoporosis. ① = Total effective rate, ② = Short Form-36, Health Survey (SF-36), ③ = Fracture healing time, ④ = Lumbar vertebra BMD ⑤ = Femoral neck BMD ⑥ = Ward’s triangle BMD, ⑦ = Serum BGP, levels ⑧ = Serum BALP, levels ⑨ = Serum TRACP-5b levels ⑩ = Adverse events.

### 3.3 Quality assessment

**FIGURE 2:**
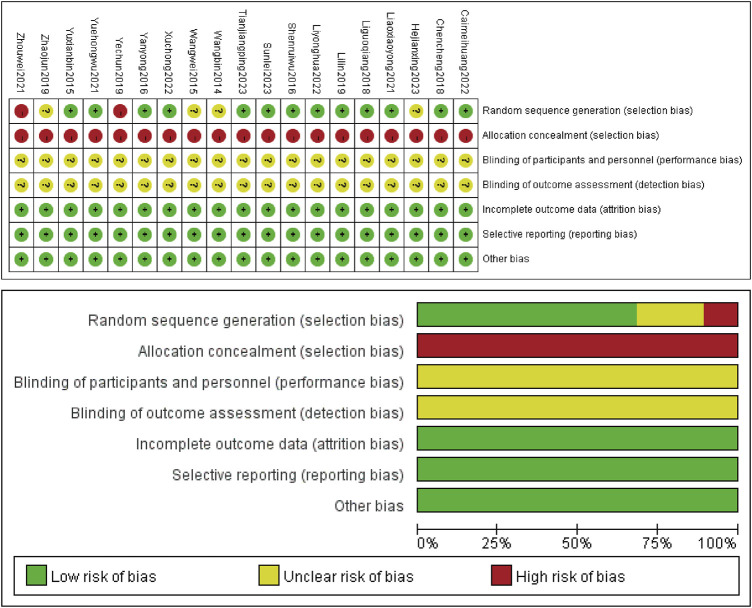
The methodological quality of the included studies.

Twelve studies (Yu et al., 2015; Shen, 2016; Yan, 2016; Chen, 2018; Li, 2018; Li et al., 2019; Yue and Huang, 2021; Cai et al., 2022; Li et al., 2022; Xu, 2022; Sun and Xie, 2023; Tian, 2023) were grouped based on the random number table method (low risk), one study (Liao et al., 2021) were grouped based on the modular arithmetic method (low risk), one study (Ye, 2019) were grouped based on treatment protocol (high risk), and one study (Zhou et al., 2021) were grouped based on single or even numbers (high risk). Four studies (Wang et al., 2014; Wang et al., 2015; Zhao et al., 2019; He et al., 2023) did not mention the specific randomized methods used (unclear). Allocation hiding (high risk) was not implemented in 19 studies (Wang et al., 2014; Wang et al., 2015; Yu et al., 2015; Shen, 2016; Yan, 2016; Chen, 2018; Li, 2018; Li et al., 2019; Ye, 2019; Zhao et al., 2019; Liao et al., 2021; Yue and Huang, 2021; Zhou et al., 2021; Cai et al., 2022; Li et al., 2022; Xu, 2022; He et al., 2023; Sun and Xie, 2023; Tian, 2023). None of the 19 studies reported whether they were blinded (unclear). All studies (Wang et al., 2014; Wang et al., 2015; Yu et al., 2015; Shen, 2016; Yan, 2016; Chen, 2018; Li, 2018; Li et al., 2019; Ye, 2019; Zhao et al., 2019; Liao et al., 2021; Yue and Huang, 2021; Zhou et al., 2021; Cai et al., 2022; Li et al., 2022; Xu, 2022; He et al., 2023; Sun and Xie, 2023; Tian, 2023) had complete data (low risk), no selective reporting of findings (low risk), and no other sources of bias (low risk) (Figure 2).

### 3.4 Results of meta-analysis

#### 3.4.1 Total effective rate

**FIGURE 3:**
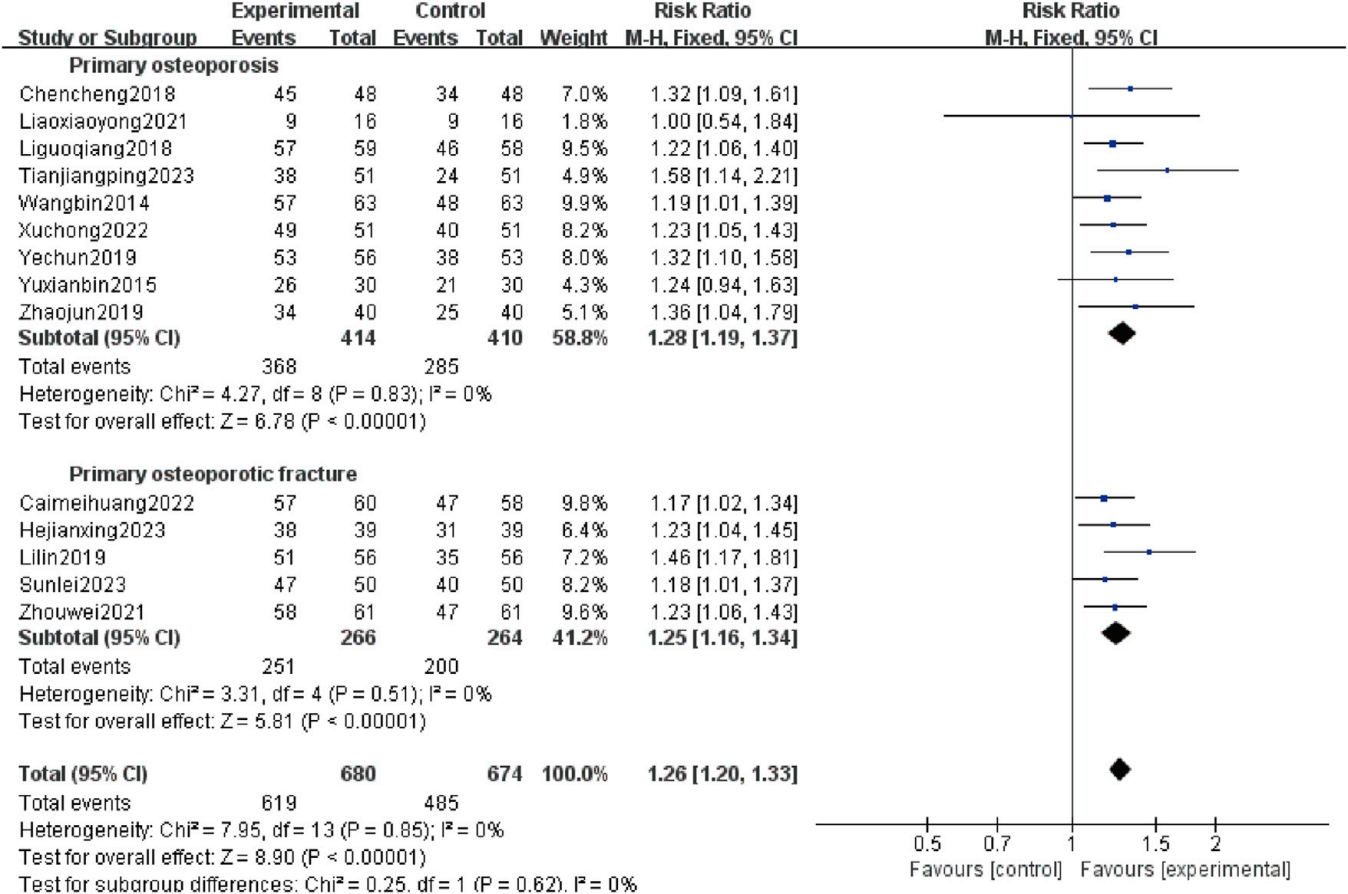
Forest plot of total effective rate.

The total effective rate was reported in 14 studies (Wang et al., 2014; Yu et al., 2015; Chen, 2018; Li, 2018; Li et al., 2019; Ye, 2019; Zhao et al., 2019; Liao et al., 2021; Zhou et al., 2021; Cai et al., 2022; Xu, 2022; He et al., 2023; Sun and Xie, 2023; Tian, 2023) involving 1,354 patients, including 824 patients with POP alone (414 patients in test group and 410 patients in control group) and 530 patients with OPF (266 patients in test group and 264 patients in control group). Heterogeneity analysis revealed good homogeneity (*p* = 0.85, *I* ^*2*^ = 0%). The fixed effect model analysis showed that the total effective rate was higher in the experimental group than the control group [*RR*:1.26; 95% *CI*:1.20 to 1.33; *p* < 0.00 001]. The results of subgroup analysis showed that regardless of whether OPF was combined, adding Gukang capsule to the original treatment in the control group could improve the total effective rate [*RR:*1.28; 95%*CI*:1.19 to 1.37; *p* < 0.00 001], and [*RR*:1.25; 95% *CI*:1.16 to 1.34; *p* < 0.00 001] (Figure 3).

#### 3.4.2 Quality of life

**FIGURE 4:**
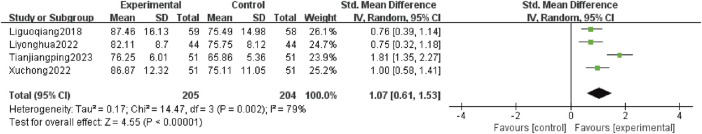
Forest plot of SF-36.

The Medical Outcomes Study Short-Form 36 (SF-36) is a general health parameter designed for use in health policy population surveys or evaluative studies. It contains 36 items covering both the physical and mental health aspects and is one of the world’s most commonly used standardized Quality of life measurement tools (Patel et al., 2007). Four studies (Li, 2018; Li et al., 2022; Xu, 2022; Tian, 2023) reported SF-36 scores. Heterogeneity analysis revealed variations (*p* = 0.002, *I* ^*2*^ = 79%). The random effects model analysis showed that adding Gukang Capsule to the original treatment in the control group could improve the SF-36 scores for POP patients [*SMD*:1.07; 95% *CI:*0.61 to 1.53; *p* < 0.00 001] as well as their QoL (Figure 4).

#### 3.4.3 Fracture healing time

**FIGURE 5:**
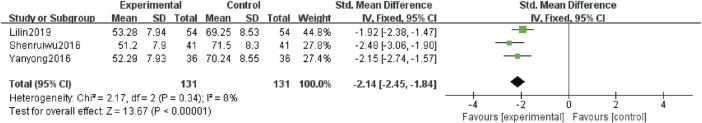
Forest plot of the fracture healing time.

Three studies (Wang et al., 2015; Shen, 2016; Yan, 2016) reported the fracture healing time. Heterogeneity analysis revealed good homogeneity (*p* = 0.34, *I* ^*2*^ = 8%). The fixed effect model analysis showed that adding Gukang Capsule to the original treatment in the control group could shorten the fracture healing time for OPF patients [*SMD*:-2.14; 95% *CI*:-2.45 to −1.84; *p* < 0.00 001] (Figure 5).

#### 3.4.4 Lumbar vertebra BMD

**FIGURE 6:**
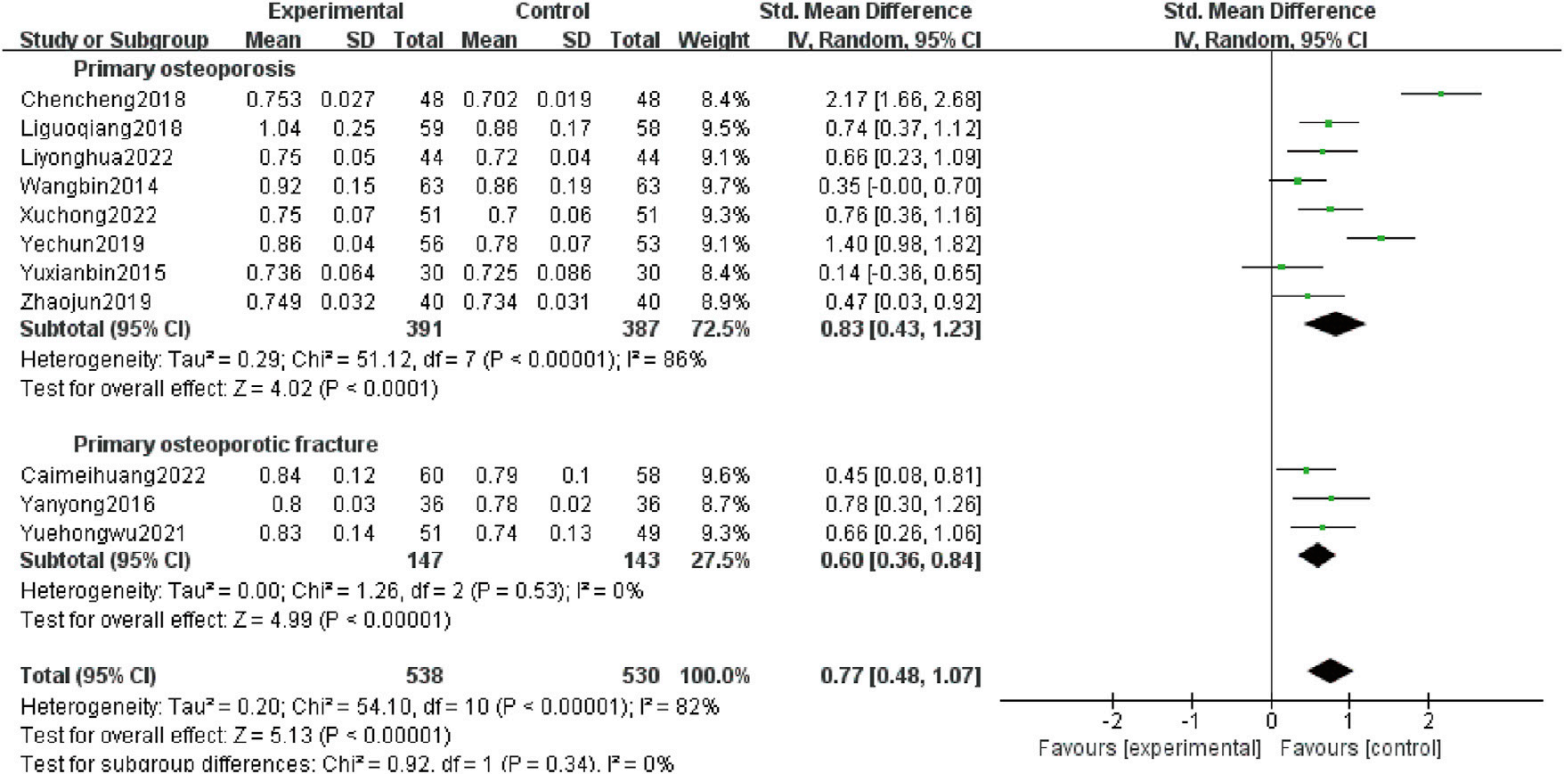
Forest plot of lumbar vertebra BMD.

Eleven studies (Wang et al., 2014; Yu et al., 2015; Yan, 2016; Chen, 2018; Li, 2018; Ye, 2019; Zhao et al., 2019; Yue and Huang, 2021; Cai et al., 2022; Li et al., 2022; Xu, 2022) reported lumbar vertebra BMD, involving 1,068 patients, including 778 patients with POP alone (391 patients in test group and 387 patients in control group) and 290 patients with OPF (147 patients in test group and 143 patients in control group). Heterogeneity analysis revealed variations (*p* < 0.00 001, *I* ^*2*^ = 82%). The random effects analysis showed that the lumbar vertebra BMD was higher in the experimental group than the control group [*SMD*:0.77; 95% *CI:*0.48 to 1.07; *p* < 0.00 001]. Subgroup analysis results showed that adding Gukang Capsule to the original treatment in the control group could improve the lumbar vertebra BMD regardless of whether POP and OPF were combined [*SMD*:0.83; 95% *CI:*0.43 to 1.23; *p* < 0.0 001], and [*SMD*:0.60; 95% *CI:*0.36 to 0.84; *p* < 0.00 001] (Figure 6).

#### 3.4.5 Femoral neck BMD

**FIGURE 7:**
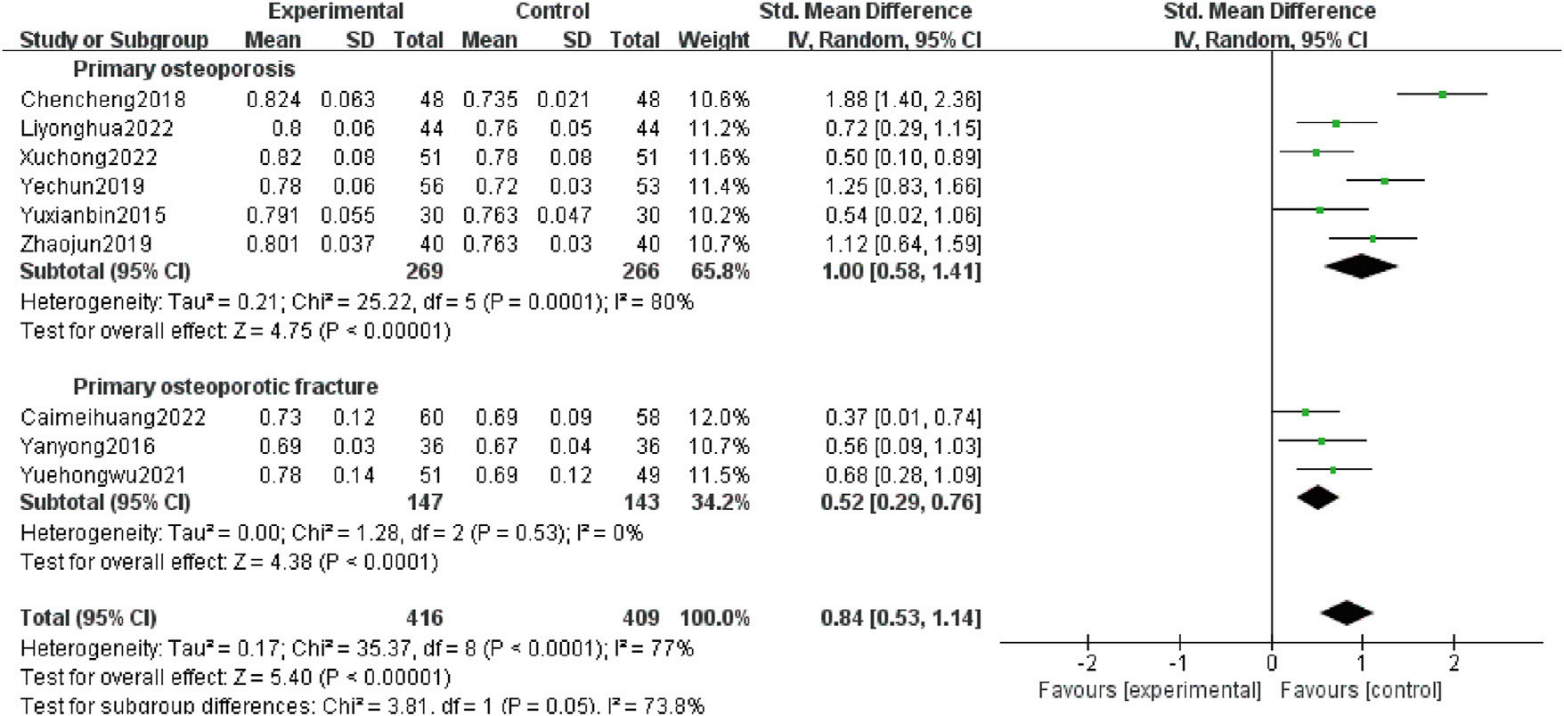
Forest plot of femoral neck BMD.

Nine studies (Yu et al., 2015; Yan, 2016; Chen, 2018; Ye, 2019; Zhao et al., 2019; Yue and Huang, 2021; Cai et al., 2022; Li et al., 2022; Xu, 2022) reported femoral neck BMD, involving 925 patients, including 535 patients with POP alone (269 patients in test group and 266 patients in control group) and 290 patients with OPF (147 patients in test group and 143 patients in control group). Heterogeneity analysis revealed variations (*p* < 0.0 001, *I* ^*2*^ = 77%). The random effects model analysis showed that the femoral neck BMD was higher in the experimental group than the control group [*SMD*:0.84; 95% *CI:*0.53 to 1.14; *p* < 0.00 001]. Subgroup analysis showed that adding Gukang Capsule to the original treatment in the control group could improve the femoral neck BMD regardless of whether POP and OPF were combined [*SMD*:1.00; 95% *CI:*0.58 to 1.41; *p* < 0.00 001], and [*SMD*:0.52; 95% *CI:*0.29 to 0.76; *p* < 0.0 001] (Figure 7).

#### 3.4.6 Ward’s triangle BMD

**FIGURE 8:**
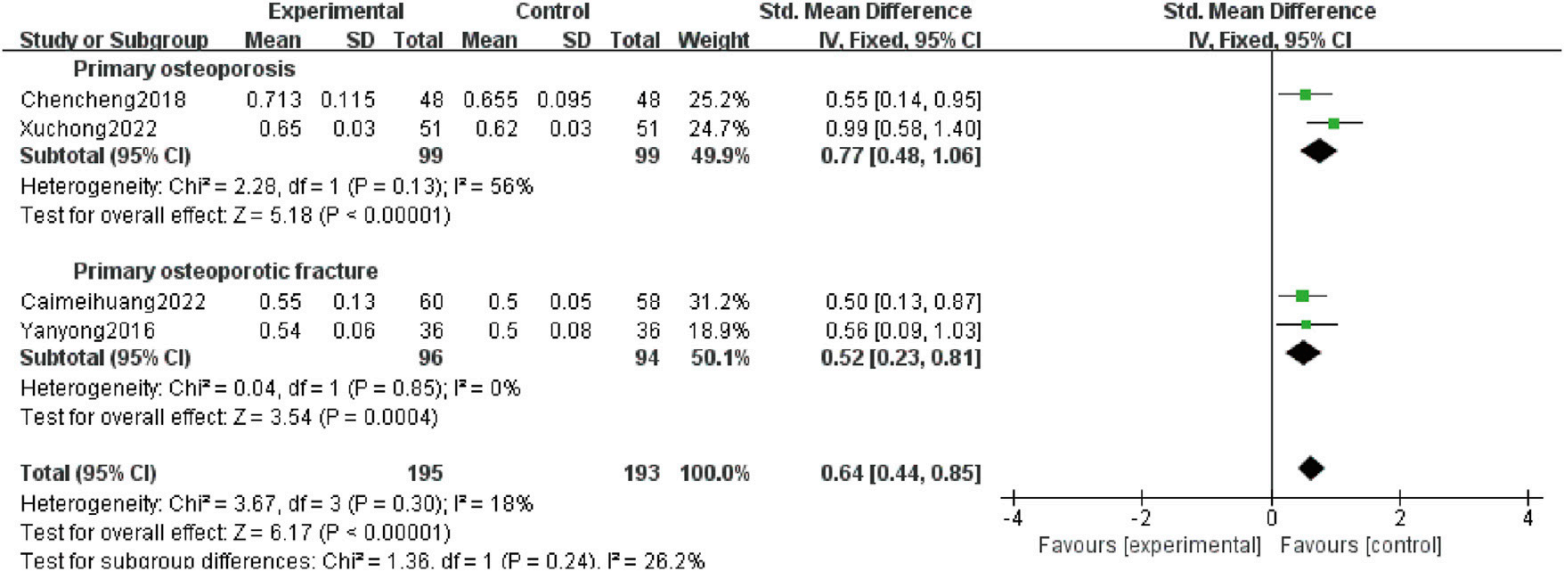
Forest plot of Ward’s triangle BMD.

Four studies (Yan, 2016; Chen, 2018; Cai et al., 2022; Xu, 2022) reported Ward’s triangle BMD, involving 388 patients, including 198 patients with POP alone (99 patients in test group and 99 patients in control group) and 190 patients with OPF (96 patients in test group and 94 patients in control group). Heterogeneity analysis revealed good homogeneity (*p* = 0.30, *I* ^*2*^ = 18%). The fixed effect model analysis showed that Ward’s triangle BMD was higher in the experimental group than the control group [SMD:0.64; 95% *CI:*0.44 to 0.85; *p* < 0.00 001]. Subgroup analysis showed that adding Gukang Capsule to the original treatment in the control group could improve Ward’s triangle BMD regardless of whether POP and OPF were combined [SMD:0.77; 95% *CI:*0.48 to 1.06; *p* < 0.00 001], and [SMD:0.52; 95% *CI:*0.23 to 0.81; *p* = 0.0004] (Figure 8).

#### 3.4.7 Serum BGP levels

**FIGURE 9:**
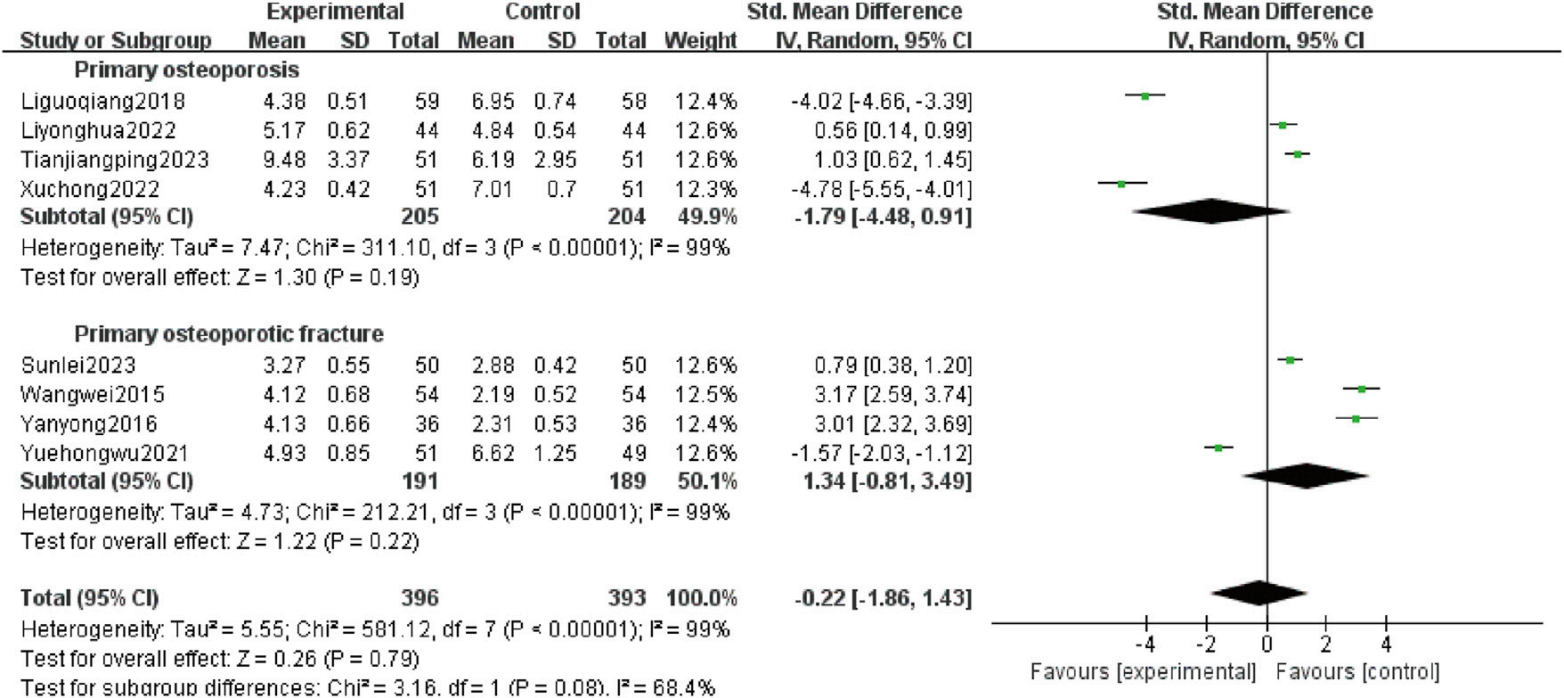
Forest plot of serum BGP levels.

The BGP is a specific biochemical index reflecting bone formation. It is involved in bone resorption regulation, matrix mineralization, and osteoblast differentiation and is related to bone turnover. Clinically, serum BGP levels are correlated with changes in osteogenic function. Anti-bone resorption drugs and bone formation stimulation therapy can decrease and increase BGP levels, respectively (Eastell and Hannon, 2008). Eight studies (Wang et al., 2015; Yan, 2016; Li, 2018; Yue and Huang, 2021; Li et al., 2022; Xu, 2022; Sun and Xie, 2023; Tian, 2023) reported serum BGP levels, involving 789 patients, including 409 patients with POP alone (205 patients in test group and 204 patients in control group) and 380 patients with OPF (191 patients in test group and 189 patients in control group). Heterogeneity analysis revealed significant variations (*p* < 0.00 001, *I* ^*2*^ = 99%). The random effects model analysis showed no difference in serum BGP levels between the experimental and control groups [*SMD*:-0.22; 95% *CI:*−1.86 to 1.43; *p* = 0.79]. Subgroup analysis showed that adding Gukang Capsule to the original treatment in the control group did not impact the serum BGP levels regardless of whether POP and OPF were combined [*SMD*:−1.79; 95% *CI*:−4.48 to 0.91; *p* = 0.19], and [*SMD*:1.34; 95% *CI:*−0.81 to 3.49; *p* = 0.22] (Figure 9).

#### 3.4.8 Serum BALP levels

**FIGURE 10:**
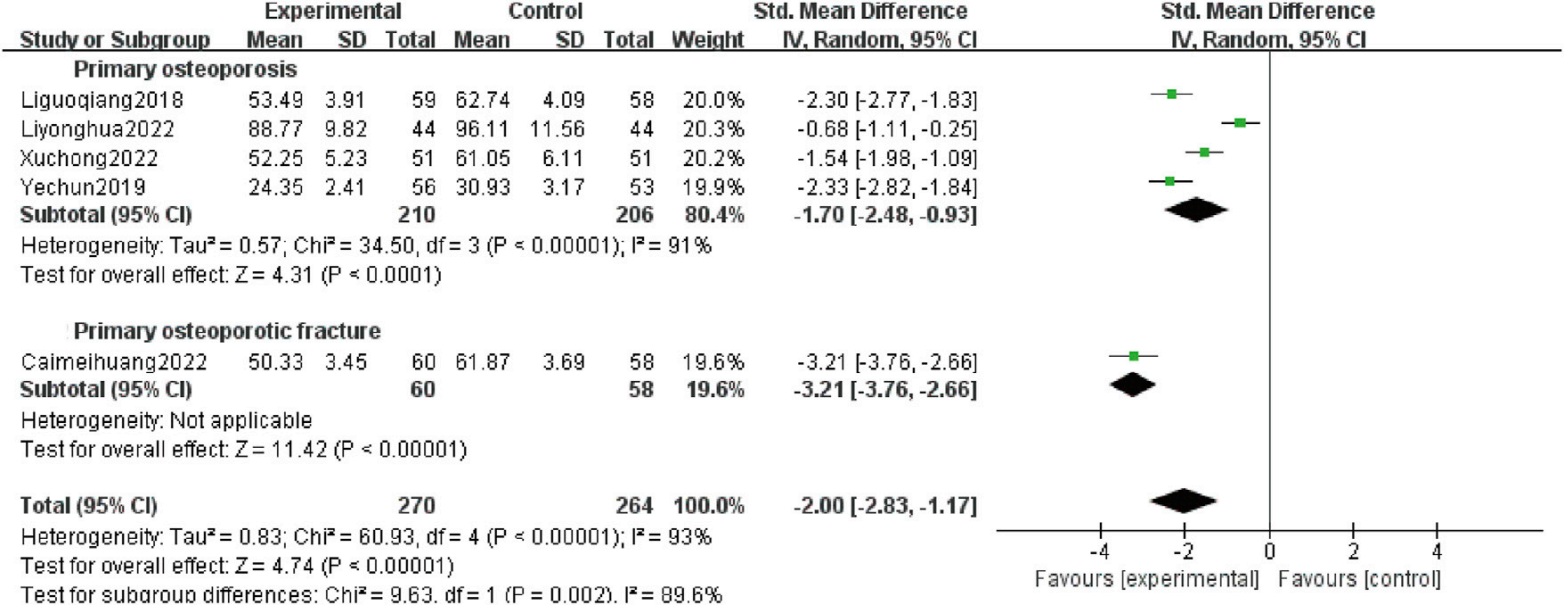
Forest plot of serum BALP levels.

The BALP is an extracellular enzyme in osteoblasts. Its primary role is to hydrolyze phosphatase during osteogenesis, providing phosphoric acid for depositing hydroxyapatite, which is conducive for bone formation. Osteoblasts synthesized a large amount of alkaline phosphatase when bone mineralization was hindered, significantly increasing the serum BALP levels (Lumachi et al., 2009). Furthermore, OP treatment with diphosphonates can decrease the BALP levels. Five studies (Li, 2018; Ye, 2019; Cai et al., 2022; Li et al., 2022; Xu, 2022) reported serum BALP levels, involving 534 patients, including 416 patients with POP alone (210 patients in test group and 206 patients in control group) and 118 patients with OPF (60 patients in test group and 58 patients in control group). Heterogeneity analysis revealed variations (*p* < 0.00 001, *I* ^*2*^ = 93%). The random effects model analysis demonstrated that the serum BALP levels were lower in the experimental group than the control group [*SMD*:−2.00; 95% *CI:*−2.83 to −1.17; *p* < 0.00 001]. Subgroup analysis showed that adding Gukang Capsule to the original treatment in the control group could reduce the serum BALP levels regardless of whether POP and OPF were combined [*SMD*:−1.70; 95% *CI:*−2.48 to −0.93; *p* < 0.0 001], and [*SMD*:−3.21; 95% *CI:*−3.76 to −2.66; *p* < 0.00 001] (Figure 10).

#### 3.4.9 Serum TRACP-5 levels

**FIGURE 11:**
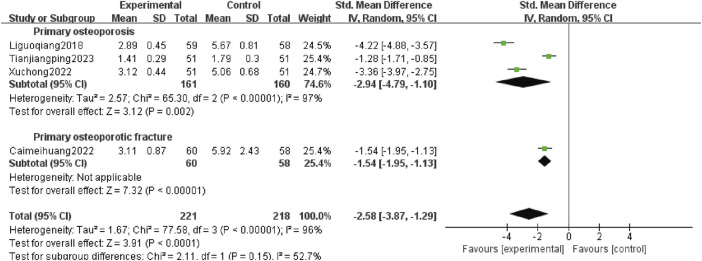
Forest plot of serum TRACP-5b levels.

The TRACP-5b is a specific and highly sensitive second-generation bone resorption marker. In POP patients, osteoclast activity was increased, bone remodeling was unbalanced, bone resorption exceeded bone formation, and the serum TRACP-5 levels were significantly increased and negatively correlated with BMD (Linossier et al., 2022). Four studies (Li, 2018; Zhao et al., 2019; Xu, 2022; Tian, 2023) reported serum TRACP-5b levels, involving 439 patients, including 321 patients with POP alone (161 patients in test group and 160 patients in control group) and 118 patients with OPF (60 patients in test group and 58 patients in control group). Heterogeneity analysis revealed variations (*p* < 0.00001, *I* ^*2*^ = 96%). The random effects model analysis showed that the serum TRACP-5b levels were lower in the experimental group than the control group [*SMD*:-2.58; 95% *CI:*−3.87 to −1.29; *p* < 0.0 001]. Subgroup analysis showed that adding Gukang Capsule to the original treatment in the control group could reduce the serum TRACP-5b levels regardless of whether POP and OPF were combined [*SMD*:−2.94; 95% *CI*:−4.79 to −1.10; *p* = 0.002], and [*SMD*:-1.54; 95% *CI*:−1.95 to −1.13; *p* < 0.00 001] (Figure 11).

#### 3.4.10 Adverse events

**FIGURE 12:**
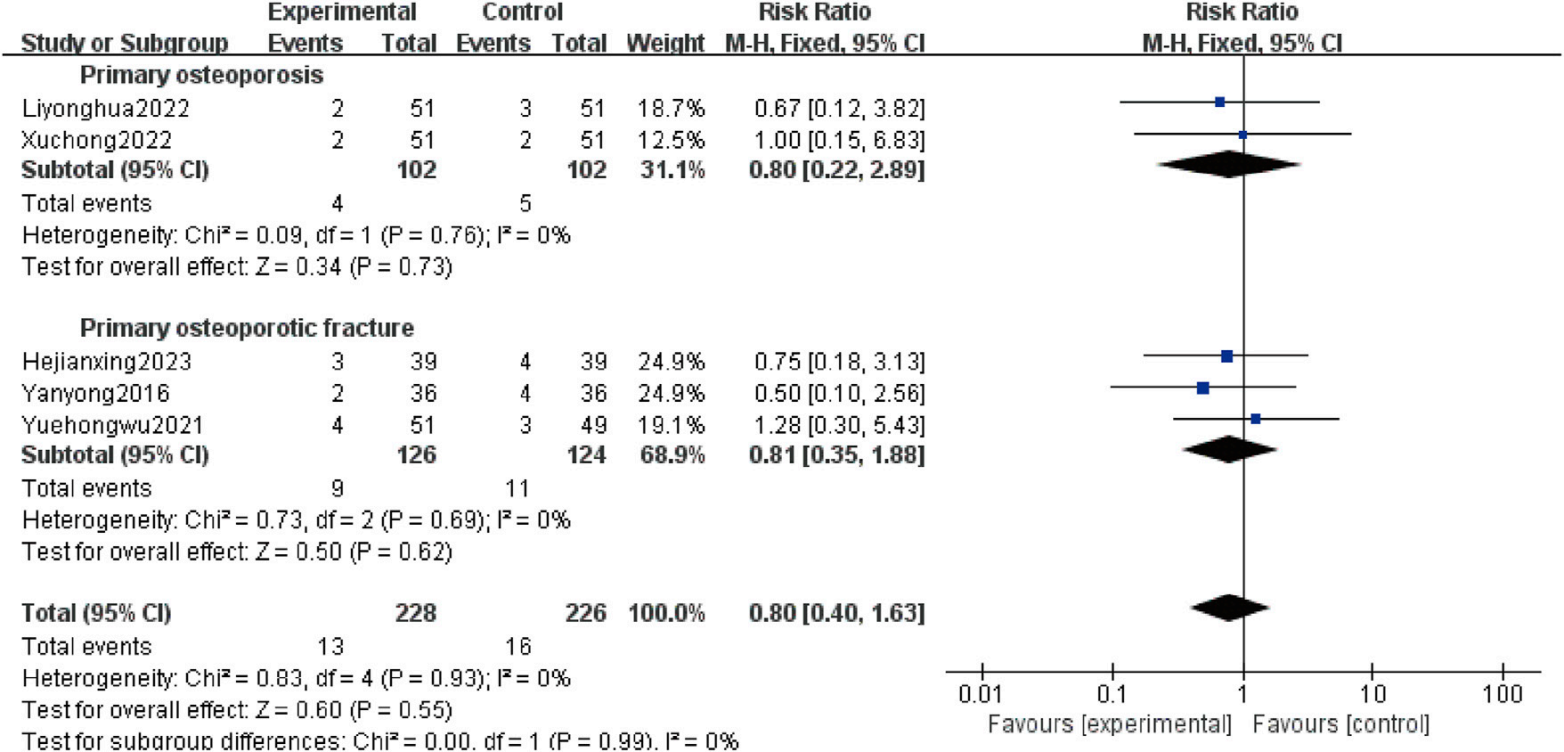
Forest plot of adverse events.

Five studies (Yan, 2016; Yue and Huang, 2021; Li et al., 2022; Xu, 2022; He et al., 2023) reported AEs, involving 454 patients, including 204 patients with POP alone (102 patients in test group and 102 patients in control group) and 250 patients with OPF (126 patients in test group and 124 patients in control group). Heterogeneity analysis revealed good homogeneity (*p* = 0.93, *I* ^*2*^ = 0%). The fixed effect model analysis revealed no difference in the AEs between the experimental and control groups [*RR*:0.80; 95% *CI*:0.40 to1.63; *p* = 0.55]. Subgroup analysis showed that adding Gukang Capsule to the original treatment in the control group had no impact on AEs regardless of whether POP and OPF were combined [*RR*:0.80; 95% *CI:*0.22 to 2.89; *p =* 0.73], and [RR:0.81; 95% *CI:*0.35 to 1.88; *p* = 0.62] (Figure 12).

### 3.5 Sensitivity analysis

**FIGURE 13:**
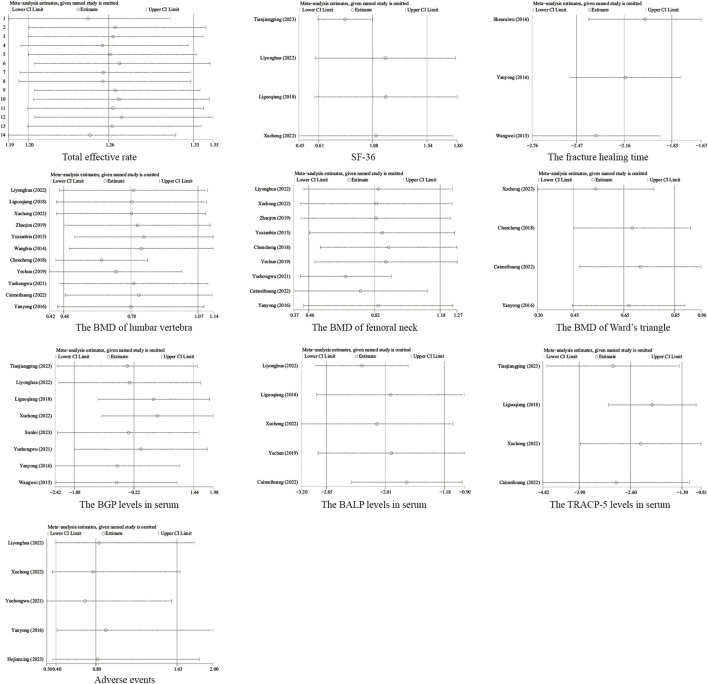
Sensitivity analysis of each outcome.

Sensitivity analysis was performed for each outcome by eliminating one study at a time and recombining the statistics. No significant change was observed in all outcomes, implying the stability of the study results (Figure 13).

### 3.6 Publication bias

**FIGURE 14:**
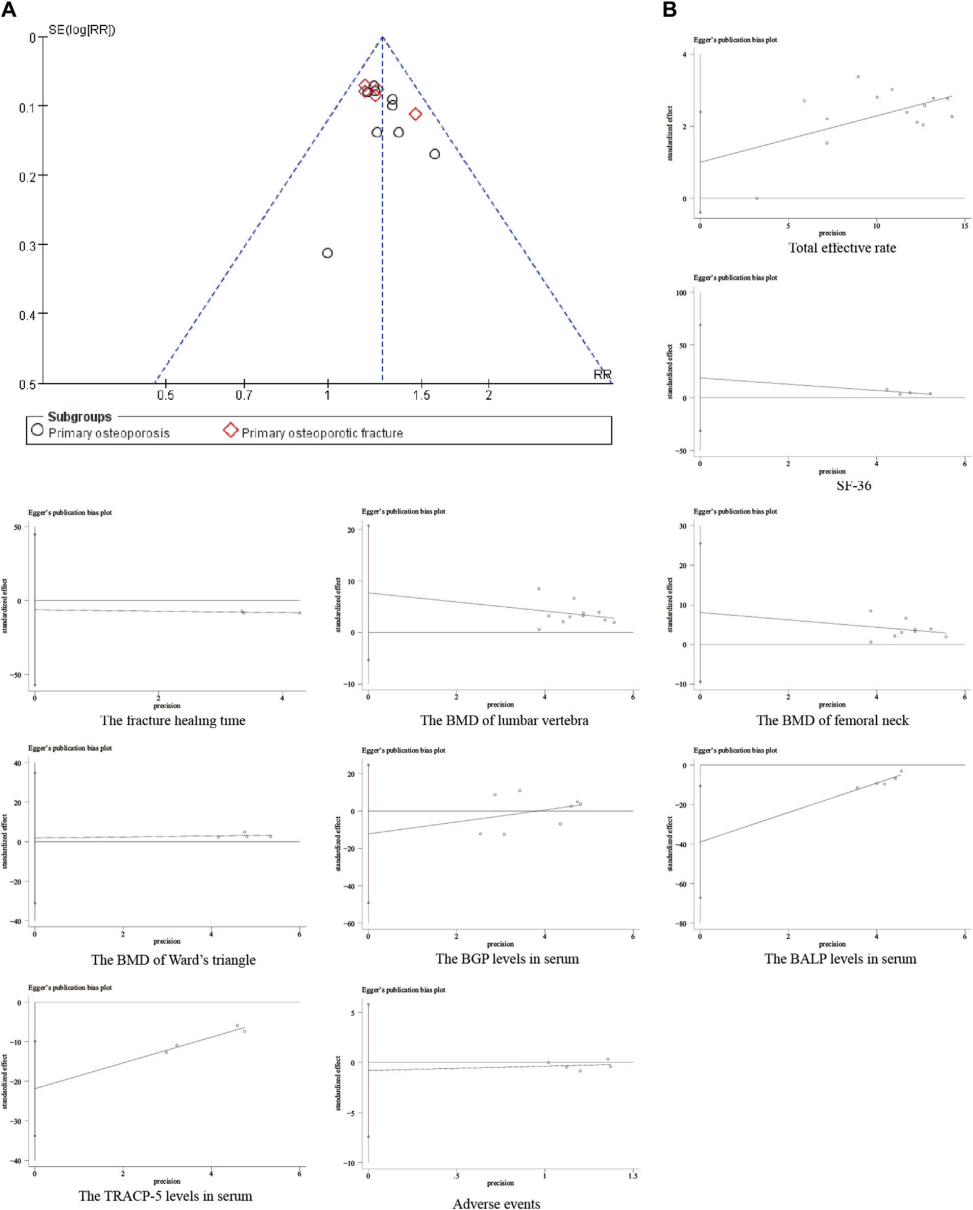
Publication bias analysis. **(A)** Funnel plot of the total effective rate; **(B)** Egger test results of each outcome.

Funnel plots are a graphical tool used to assess publication bias in a meta-analysis. They depict the relationship between the effect size (strength of the observed effect) of individual studies and their standard error (a measure of the study’s precision). In the absence of publication bias, the plot ideally resembles a symmetrical inverted funnel. Smaller studies, typically with higher standard errors, would be scattered at the bottom, while larger and more precise studies would cluster towards the top. Deviations from this symmetrical shape, particularly a narrower funnel at the bottom, can suggest publication bias. This bias occurs when studies with statistically non-significant results (those that do not show a strong effect) are less likely to be published, leading to an overestimation of the overall treatment effect (Hopp, 2015). The publication bias funnel plot was drawn using the total effective rate. The distribution of scattered points in each study was asymmetrical, implying potential publication bias (Figure 14A). Egger’s test is a statistical test used to quantify the funnel plot asymmetry and provide a more objective assessment of publication bias. A significant result from Egger’s test suggests the presence of publication bias (Hopp, 2015). The Egger’s test was used to quantify each outcome’s bias. According to the results, the P>|t|-values of serum BALP levels and serum TRACP-5b levels were <0.1, indicating potential publication bias. The *P*>|t|-values of the other outcomes were >0.1, implying no significant bias. Publication bias may be the result of concealing negative results. (Figure 14B). Since positive results are more likely to be published and cited, negative results are often overlooked or not readily available, which may lead to the bias of meta-analysis results.

## 4 Discussion

Bone mass naturally declines with age as part of the ongoing physiological processes of growth, development, and aging. This leads to an acceleration in bone turnover, resulting in increased bone loss. OPFs are a major complication of OP and the most evident consequence of this decline in bone strength. Notably, OPFs most commonly occur in the vertebrae, hip, and distal radius (Spiegl et al., 2021). Although OP can be managed through lifestyle practices such as exercises and sufficient intake of calcium and vitamin D, medications have been developed for the treatment of OP. Given its multi-component, multi-target, high safety, and unique advantages in treating chronic illnesses and complications, TCM has gradually become a Complementary and Alternative Medicine (CAM) for OP treatment (Li et al., 2022; Jia et al., 2023). Gukang Capsule is a common Chinese patent medicine for clinically treating OP in China. The main Gukang Capsule ingredients include Rhizoma musae [Musaceae; Musa basjoo], Notoginseng radix et rhizoma [Araliaceae; Panax Notoginseng (Burk.) F. H. Chen Ex C. Chow], Oxalis corniculata L. [Oxalidaceae; Oxalis lata L.], Psoraleae fructu [Leguminosae; Psoralea corylifolia L.] and Dipsaci radix [Dipsacaceae; Dipsacus asper Wall. ex Henry].

### 4.1 Effectiveness of GC in treating POP

Yang et al. (2018) explored the Gukang Capsule effects on the proliferation, differentiation, and mineralization of human osteoblast SaOS-2 cultured *in vitro*. The results showed that GC can promote osteoblast proliferation, differentiation, and mineralization. Furthermore, a literature search revealed that Gukang Capsule can be used in combination with calcium, vitamin D, bisphosphonates, or calcitonin in POP treatment. Overall, Gukang Capsule has been reported in several studies to exert a good curative effect in POP patients. However, the conclusions of these studies are often not evidence-based and unconvincing due to the influence of inconsistent treatment methods, and insufficient sample sizes. Herein, 19 RCTs (1804 patients) were reviewed to further elucidate Gukang Capsule efficacy in treating POP. Compared to the control group, treatment with Gukang Capsule resulted in several positive outcomes. These included increased BMD in the lumbar vertebrae, femoral head, and Ward’s triangle, improved quality of life, shortened healing time for OPFs, and a higher total effective rate. Furthermore, a meta-analysis of serum bone metabolism markers revealed that Gukang Capsule may regulate bone formation and resorption. This is evidenced by reductions in levels of bone ALP (BALP) and TRACP-5b, which are indicators of these processes.

The Standardization Office of the Chinese Society of Traditional Chinese Medicine systematically combed the clinical and basic research results of Gukang Capsule since its listing, and formed the ‘Expert Consensus on the clinical application of Gukang Capsule in the Treatment of Osteoporosis’ on the basis of full consideration of clinical research evidence and expert experience. This consensus statement outlines the recommended use of Gukang Capsule for treating OP. It defines the ideal patient characteristics, typical symptoms, and disease stages suitable for Gukang Capsule therapy. Moreover, it specifies the recommended dosage, treatment duration, potential interactions with other medications, and any necessary precautions for safe use. Furthermore, the statement clarifies when Gukang Capsule is contraindicated (Zhu et al., 2022). The consensus points out that Gukang capsule in the treatment of OP can improve the bone mineral density of patients (strongly recommendation), improve the level of bone turnover markers (β-CTX, PINP, N-MID-OT, 25(OH)D) and bone metabolism biochemical indicators (BGP, BALP, TRACP-5b) of patients (weak recommendation). Gukang capsule in the treatment of osteoporosis can relieve pain, improve dysfunction, improve patients’ quality of life (strongly recommended). When Gukang capsule is used in combination with other drugs, the therapeutic effect is more significant (strongly recommended) (Zhu et al., 2022).

### 4.2 Safety of Gukang capsule in treating POP

An acute drug toxicity test and long-term drug experiment showed that Gukang Capsule did not pose an acute toxicity risk or delay drug toxicity (Zhu et al., 2022). Additionally, a study was conducted on the effect of Gukang Capsule on drug transporter protein expression in rat liver. The results showed that Gukang Capsule could alter the expression of liver drug transporter proteins, including OATP1B1, OCT1, MRP1, MRP2, BCRP, and PGP, in a dose-dependent manner (Li et al., 2021). Combining Gukang Capsule with certain medications, particularly those with a narrow therapeutic window, may lead to drug interactions. These interactions may occur through transporter proteins and could affect how the medications are absorbed and distributed in the body. Zhu et al. (Zhu et al., 2018) screened literature reports of cases and groups of cases of Gukang Capsule-induced liver injury, as well as literature reports of adverse reaction monitoring data after marketing, and analyzed pertinent cases in the literature. According to the results, liver injury was the main adverse reaction to Gukang Capsule, with the risk being primarily related to psoralen. Long-term Gukang Capsule intake, Gukang Capsule use in elderly patients, and liver disease history may be the pre-risk factors for liver injury. Five clinical studies included in this analysis reported adverse reactions associated with drug therapy. However, the meta-analysis revealed no statistically significant difference in the frequency of adverse reactions between the Gukang Capsule group and the control group. Furthermore, no serious side effects were reported, and the most common adverse events were mild gastrointestinal issues such as nausea and diarrhea. These findings suggest that Gukang Capsule does not appear to increase the risk of side effects and may be a relatively safe option as a complementary or alternative treatment for POP.

The ‘Consensus’ suggests that Gukang capsule for the treatment of OP, if there is a combination of drugs should be cautious, should not be combined with hepatotoxic drugs. The consensus adverse reactions of taking Gukang capsule were summarized. Adverse reactions of digestive system included: nausea, vomiting, poor appetite, gastrointestinal discomfort, abdominal pain, diarrhea, abdominal distension, constipation, abnormal liver biochemical indexes, etc. Skin and accessory adverse reactions include: rash, pruritus, etc. Other adverse reactions include dizziness, headache, fever, fatigue, and dark urine color. It is recommended to take Gukang capsule after meals, stop taking medicine immediately when adverse reactions occur, go to relevant departments for diagnosis and treatment, and recommend liver function examination (Zhu et al., 2022).

### 4.3 Limitations

This study has the following limitations. (1) None of the studies employed double-blinding or allocation concealment, and overall study designs lacked rigor. This raises concerns about potential bias in the results. (2) The studies used a variety of drug combinations and treatment durations. This inconsistency makes it difficult to establish a standardized treatment regimen for Gukang Capsule. (3) Evaluating the long-term effectiveness and safety of Gukang Capsule requires extended follow-up data, which was missing in many of the included studies. (4) The studies were limited to China, as Gukang Capsule is not standardized in other countries. This geographic limitation may introduce publication bias. (5) Positive results are generally more likely to be published and cited than negative findings. This can lead to biased conclusions in meta-analysis.

## 5 Conclusion

This meta-analysis suggests that Gukang Capsule may be a promising CAM for POP due to its observed clinical efficacy and safety. However, the quality limitations of the included studies weaken the strength of this conclusion. To generate more reliable evidence, well-designed, multicenter RCTs with large sample sizes are necessary. Future research should also explore the cost-effectiveness of Gukang Capsule in POP treatment. Evaluating its economic advantages can clarify its value as a CAM and inform decisions about resource allocation within healthcare systems.

## Data Availability

The original contributions presented in the study are included in the article/Supplementary Material, further inquiries can be directed to the corresponding author.
