# Supplementary material for: The efficacy and safety of Gukang Capsule for primary osteoporosis: a systematic review and meta-analysis of randomized clinical trial
Source: Front Pharmacol. 2024 Jun 10;15:1394537. doi: 10.3389/fphar.2024.1394537 (PMC11194336; doi:10.3389/fphar.2024.1394537)
Supplement: Supplementary file 1 [file DataSheet1.zip › Supplementary File S1.DOCX]

**Supplementary File S1. Composition of the Gukang capsule**

| **Chinese drug name** | **Latin name** | **Botanical plant name** | **Family** | **Plant part used** |
| --- | --- | --- | --- | --- |
| Bajiaogen | Rhizoma musae | Musa basjoo | Musaceae | Root and rhizome |
| Sanqi | Notoginseng radix et rhizoma | Panax Notoginseng (Burk.) F. H. Chen Ex C. Chow | Araliaceae | Root and rhizome |
| Cujiangcao | Oxalis corniculata L. | Oxalis lata L. | Oxalidaceae | whole herb |
| Buguzhi | Psoraleae fructu | Psoralea corylifolia L. | Leguminosae | Ripe fruit |
| Xuduan | Dipsaci radix | Dipsacus asper Wall. ex Henry | Dipsacaceae | Root and rhizome |
