# Supplementary material for: The efficacy and safety of Gukang Capsule for primary osteoporosis: a systematic review and meta-analysis of randomized clinical trial
Source: Front Pharmacol. 2024 Jun 10;15:1394537. doi: 10.3389/fphar.2024.1394537 (PMC11194336; doi:10.3389/fphar.2024.1394537)
Supplement: Supplementary file 1 [file DataSheet1.zip › Supplementary File S2.DOCX]

**Supplementary File S2. Extract and extraction process description of the Gukang capsule.**

The extract and extraction process descripyion of the Gukang capsule are as follows:

(1) According to the preparation of 1000 grains, the dosage of its components is: 600g of Rhizoma musae, 400g of Oxalis corniculata L., 600g of Psoraleae fructu, 300g of Dipsaci radix, and 150g of Notoginseng radix et rhizoma.

(2) First, Pulverize the Notoginseng radix et rhizoma, pass the 100-mesh sieve, and then pack it and seal it for use;

(3) Cook Rhizoma musae, Oxalis corniculata L., Psoraleae fructu and Dipsaci radix with water for 3 times, 2 hours for the first time, 1.5 hours for the second time, and 1 hour for the third time, combine the 3 times of liquid, concentrate into paste, the relative density is 1.31~1.35, dry the paste, and grind it through a 100-mesh-sieve.

(4) Mix the above powder with panax Notoginseng powder, and then fill the capsule.

References

1. He SM. Gukang capsule and its production technology. China. Patent No CN03101236.1. State Intellectual Property Office of the People's Republic of China.
