# Supplementary material for: The efficacy and safety of Gukang Capsule for primary osteoporosis: a systematic review and meta-analysis of randomized clinical trial
Source: Front Pharmacol. 2024 Jun 10;15:1394537. doi: 10.3389/fphar.2024.1394537 (PMC11194336; doi:10.3389/fphar.2024.1394537)
Supplement: Supplementary file 1 [file DataSheet1.zip › Supplementary File S3.DOCX]

**Supplementary File S3. The identification process of** **Gukang** **capsule.**

**The identification process of** **Gukang capsule is as follows:****^[1.2]^**

1. Take 5g of Gukang capsule, add 30ml of methanol, ultrasound process for 30 minutes, filter, concentrate the filtrate to 1ml as the test sample solution; take 1g of Rhizoma musae as the control herbal medicine, and prepare the control herbal medicine solution using the same method; conduct thin-layer chromatography experiment, take 5μl of each of the above two solutions, spot them on the same silica gel G thin-layer plate, use petroleum ether (60-90°C): ethyl acetate (20:1) as the developing agent, develop, remove, air-dry, spray with 10% sulfuric acid ethanol solution, heat to 105°C until the spots are clearly colored, and observe under daylight and ultraviolet lamp (365nm) respectively; in the test sample chromatography, at the corresponding positions to the control herbal medicine chromatography, the same colored spots or fluorescent spots appear.
2. Take 5g of Gukang capsule, add 40ml of methanol, reflux for 1 hour, filter, evaporate the filtrate, dissolve the residue in 15ml of water, extract with 20ml of chloroform by shaking, discard the chloroform layer, extract the aqueous solution with water-saturated n-butanol twice (20ml, 15ml), combine the n-butanol layers, wash with ammonia solution twice (30ml, 15ml), discard the ammonia solution, evaporate the n-butanol layer, dissolve the residue in 1ml of methanol as the test sample solution; take 1g of Notoginseng radix et rhizoma as the control herbal medicine, add 20ml of methanol, prepare the control herbal medicine solution using the same method; then take ginsenoside R1 and ginsenoside Rg1 reference standards, dissolve each in methanol to a concentration of 2mg/ml, as the reference solution; conduct thin-layer chromatography experiment, spot 2-5μl of each of the three solutions on the same silica gel G thin-layer plate, use the lower layer solution of chloroform: ethyl acetate: methanol: water (15:40:22:10) stored at below 10°C overnight as the developing agent, develop, remove, air-dry, spray with 10% sulfuric acid ethanol solution, heat to 105°C until the spots are clearly colored; in the test sample chromatography, spots of the same color appear at corresponding positions to the control herbal medicine and reference standards chromatograms.

**The fingerprint chromatographic method for the detection of Gukang medicine is as follows: ^[1]^**

Using high-performance liquid chromatography with octadecylsilane-bonded silica gel as the stationary phase; acetonitrile as mobile phase A and 0.1% phosphoric acid solution as mobile phase B for gradient elution. The elution method is as follows: use 10% mobile phase A and 90% mobile phase B from 0 to 10 minutes, gradient from 10% to 30% mobile phase A and from 90% to 70% mobile phase B from 10 to 30 minutes, from 30% to 50% mobile phase A and from 70% to 50% mobile phase B from 30 to 60 minutes, from 50% to 70% mobile phase A and from 50% to 30% mobile phase B from 60 to 100 minutes; the flow rate is 1.0ml/min; detection wavelength is 330nm; the theoretical plate number for Hesperidin-6-C-β-D-Glucoside shall not be less than 5000; preparation of reference solution: take an appropriate amount of Hesperidin-6-C-β-D-Glucoside reference standard, precisely weigh, dissolve in methanol to obtain a solution containing 0.05mg of Hesperidin-6-C-β-D-Glucoside per 1ml; preparation of test sample solution: take 1.0g of Guikang medicine content, precisely weigh, place in a stoppered conical flask, add 25ml of methanol, weigh, sonicate for 30 minutes, cool, make up for the lost weight with methanol, filter, collect the filtrate; determination: precisely take 10μl of both the reference solution and the test sample solution, inject into the liquid chromatograph, and measure. This detection method is applicable to Gukang capsule, tablets, granules, pills, injections, decoctions, syrups, mixtures, wines, pills, and powders.

References

1. He YD, He HH, He LF, et al. Methods for the detection of Gukang drugs. China. Patent No CN201310382486.5. State Intellectual Property Office of the People's Republic of China.
2. He YD, He HH, He LF, et al. Method of quality control of Gukang drugs. China. Patent No CN201310382486.5. State Intellectual Property Office of the People's Republic of China.
