# Supplementary material for: The efficacy and safety of Gukang Capsule for primary osteoporosis: a systematic review and meta-analysis of randomized clinical trial
Source: Front Pharmacol. 2024 Jun 10;15:1394537. doi: 10.3389/fphar.2024.1394537 (PMC11194336; doi:10.3389/fphar.2024.1394537)
Supplement: Supplementary file 1 [file DataSheet1.zip › Supplementary File S4.DOCX]

**Supplementary File S4. Details about the product information of Guakang capsule**

| Product name | Ingredient | Drug character | Specification | Executive standard | Approval number | Pharmaceutical manufactories |
| --- | --- | --- | --- | --- | --- | --- |
| Guakang capsule | Rhizoma musae、Notoginseng radix et rhizoma、Oxalis corniculata L.、Psoraleae fructu、Dipsaci radix | This product is a hard capsule, the content is yellow brown powder; The smell is slight and the taste is bitter. | 0.4g per grain | China State Food and Drug Administration national drug standards WS-10464(ZD-0464)-2005-2012Z | National medicine approval code Z20025657 | Guizhou Weikang Zifan Pharmaceutical Co., LTD |

| 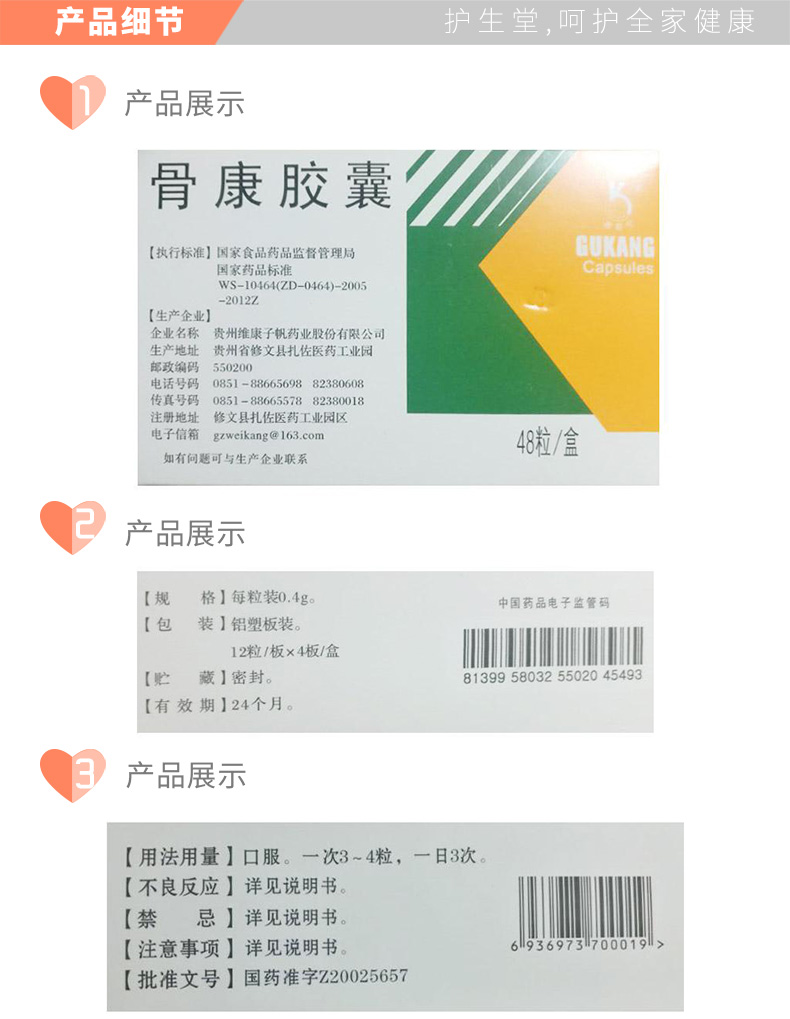 | 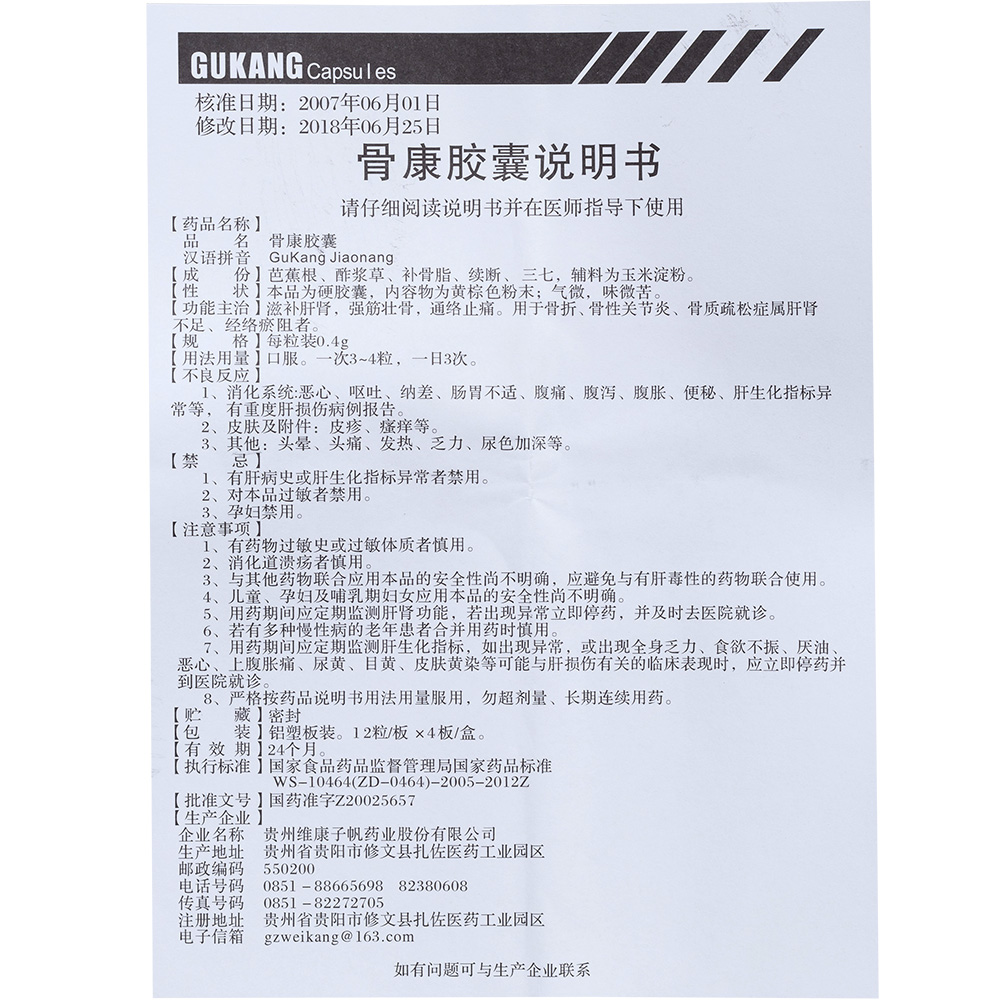 |
| --- | --- |
| Figure 1. Gukang capsule product display | Figure 2. Instructions for Gukang capsule |
