# Supplementary material for: The efficacy and safety of Gukang Capsule for primary osteoporosis: a systematic review and meta-analysis of randomized clinical trial
Source: Front Pharmacol. 2024 Jun 10;15:1394537. doi: 10.3389/fphar.2024.1394537 (PMC11194336; doi:10.3389/fphar.2024.1394537)
Supplement: Supplementary file 1 [file DataSheet1.zip › Supplementary File S5.DOCX]

**Supplementary File S5. Full Search Strategy and Results**

| Database  (Search Date) | Search Terms | Filters | Number  of results |
| --- | --- | --- | --- |
| Pubmed  (30/9/2023) | #1 (((Osteoporosis[Title/Abstract]) OR (Osteoporosis, Postmenopausal[Title/Abstract])) OR (Senile Osteoporosis[Title/Abstract])) OR (Fracture[Title/Abstract])  #2 (Gukang Capsule[Title/Abstract]) OR (Gukang Jiaonang[Title/Abstract])  #3 #1 and #2 | Language:  English | 2 |
| Web of science  (30/9/2023) | #1 Topic: (((TS=(Osteoporosis)) OR TS=(Osteoporosis, Postmenopausal)) OR TS=(Senile Osteoporosis)) AND TS=(Fracture)  #2 Topic: ((TS=(Gukang Capsule)) OR TS=(Gukang Jiaonang))  #3 #1 AND #2 | Language:  English | 9 |
| Cochrane Library  (30/9/2023) | #1 (Osteoporosis):ti,ab,kw OR (0steoporosis, Postmenopausal):t,ab,kw OR (Senile Osteoporosis):ti,ab,kw OR (Fracture):t,ab,kw  #2 (Gukang Capsule):ti,ab,kw OR (Gukang Jiaonang):ti,ab,kw  #3 #1 and #2 | Language:  English | 2 |
| 中国知网 （Chinese National Knowledge Infrastructure ）  (30/9/2023) | #1 （主题：骨康胶囊(精确)）  #2 （主题：骨质疏松症(精确)）OR（主题：原发性骨质疏松(精确)）OR（主题：老年性骨质疏松(精确)）OR（主题：绝经后骨质疏松(精确)）OR（主题：骨质疏松性骨折(精确)）  #3 #1 and #2 | Language:  Chinese | 68 |
| 维普（Chongqing VIP Information）  (30/9/2023) | #1 ((((((((题名或关键词=骨质疏松症 OR 题名或关键词=原发性骨质疏松)) OR 题名或关键词=绝经后骨质疏松)) OR 题名或关键词=老年性骨质疏松)) OR 题名或关键词=骨质疏松性骨质))  #2 题名或关键词=骨康胶囊  #3 #1 and #2 | Language:  Chinese | 31 |
| 万方（Wanfang）  (30/9/2023) | #1 主题:(骨康胶囊)  #2 (主题:(骨质疏松) or 主题:(原发性骨质疏松) or 主题:(绝经后骨质疏松) or 主题:(老年性骨质疏松) or 主题:(骨质疏松性骨折))  #3 #1 and #2 | Language:  Chinese | 68 |
